# The Presence of Stimulant Drugs in Wastewater from Krakow (Poland): A Snapshot

**DOI:** 10.1007/s00128-016-1869-5

**Published:** 2016-06-30

**Authors:** Katarzyna Styszko, Agnieszka Dudarska, Dariusz Zuba

**Affiliations:** 1 Department of Coal Chemistry and Environmental Sciences, AGH University of Science and Technology, Al. Mickiewicza 30, 30-059 Krakow, Poland; 2 Institute of Forensic Research, Westerplatte 9, 31-033 Krakow, Poland

**Keywords:** LC–ESI–QTOF–MS, Wastewater analysis, Solid phase extraction, Illicit drugs

## Abstract

An analysis of wastewater from Krakow (Poland) for the presence of controlled and uncontrolled stimulant drugs of abuse was performed. Samples were collected from the Plaszow wastewater treatment plant, Krakow, Poland, and prepared by solid phase extraction. The LC-QTOFMS method was applied for identification and quantification of popular stimulants: MDMA, mephedrone, 4-MEC, MDPV and mCPP. Environmental loads of illicit drugs were calculated; the WWTP discharged loads ranging from 3.6 to 6.7 mg day^−1^ 1000 inhabitants^−1^ of MDMA, 3.6 to 7.1 mg day^−1^ 1000 inhabitants^−1^ of mephedrone and 4.8 to 5.8 mg day^−1^ 1000 inhabitants^−1^ of 4-MEC. The results confirmed the growing popularity of new psychoactive substances in Poland.

In recent years, a large number of new psychoactive substances (NPS) have been marketed (Adamowicz et al. 2013; Zuba et al. 2013; Reid et al. 2014; Castiglioni et al. 2015; Report 2015). The first ‘head shops’ offering NPS were opened in Poland in 2008 and in 2010, around 1500 stores selling NPS without any control were in operation (Report 2011). Up to May 2009, the most popular class of substances sold as ‘legal highs’ were piperazines, including benzylpiperazine (BZP), 1-[3-trifluoromethyl)phenyl]piperazine (TFMPP), 1-(4-fluorophenyl)piperazine (pFPP), and 1-(3-chlorophenyl)piperazine (mCPP) (Byrska et al. 2010). BZP and mCPP seemed to be the most popular and easy accessible substances for users, therefore the European Monitoring Centre for Drugs and Drug Addiction (EMCDDA) carried out a risk assessment on these substances and noted drug addiction, many intoxications, including lethal poisonings. Based on their reports, BZP was banned in almost every European country (including Poland), whereas mCPP has been actively monitored (Annual Report 2012). After the ban for BZP, the market moved to a new direction; the first derivatives of cathinone were marketed (Zuba and Byrska 2013). Mephedrone (4-methylmethcathinone, MPD) became the substance of preference by users and its popularity has been growing month by month (Zuba 2014). The popularity of mephedrone was reflected in the increase in the number of drug addicts. Due to the growing popularity of NPS, monitoring of drugs of abuse in wastewater had to be expanded in order to cover a broader range of substances. A number of papers on quantification of opioids, cannabis derivatives, codeine, methadone, BZP and mCPP in wastewater were published (Zuccato et al. 2008; Baker et al. 2012; Thomas et al. 2012; Andres-Costa et al. 2014; Bijlsma et al. 2014). It was shown that due to the poor degree of purification in treatment plants, illicit drugs are still present in effluents being discharged to surface water (Kasprzyk-Hordern et al. 2009; van Nuijs et al. 2009; Zuccato and Castiglioni 2009; Mendoza et al. 2014). Due to their properties, they can be toxic to aquatic organisms (Pomati et al. 2007; Rosi-Marshall et al. 2015). Psychoactive substances have been also identified in drinking water, even after the treatment process (Castiglioni et al. 2011; Mendoza et al. 2014). Therefore, monitoring of their presence in different kinds of water is an important issue.

The aims of this pilot study were to investigate the profile of stimulant drugs taken by users in Krakow, and to estimate the environmental loads and consumption. The study covered ‘traditional’ drug of abuse, MDMA, and common novel psychoactive substances, that is mCPP, mephedrone, 4-MEC and MDPV. This is the first study based on the prevalence of stimulant drugs in the Krakow area.

## Materials and Methods

Standard solutions of mephedrone and MDPV were purchased from the Australian Government National Measurement Institute (North Ryde, Australia), MDMA from Cerilliant (Round Rock, TX, USA), mCPP from Lipomed AG (Arlesheim, Switzerland), while 4-MEC from LGC Standards Sp. z o.o. (Dziekanów Leśny, Poland). The molecular formulas, physical and chemical properties of the compounds are summarized in Table 1. The isotope labelled standard MDMA-D5 (1.0 mg mL^−1^ in methanol) was purchased from Cerilliant (Round Rock, TX, USA). HPLC supergradient grade methanol and ammonia (25 %) were obtained from POCH (Gliwice, Poland). Hydrochloric acid (32 %) and formic acid (89–91 %) were purchased from Merck (Darmstadt, Germany). HPLC-grade acetonitrile was purchased from J.T. Baker (Phillipsburg, NJ, USA). Deionized water was obtained by reverse diffusion in a Millipore system (Warsaw, Poland).

**Table 1:** List of analytes and their properties

Common name (Acronym)	Systematic name	Formulae	Log K_OW_^a^	Usage
mCPP	1-(3-Chlorophenyl)piperazine		2.07	Stimulant
4-MEC	2-(Ethylamino)-1-(4-methylphenyl)propan-1-one		2.39	Stimulant Euphoric Emphatogen
MDPV	1-(1,3-Benzodioxol-5-yl)-2-(pyrrolidin-1-yl)pentan-1-one		3.06	Stimulant Aphrodisiac
Mephedrone (MPD)	2-(Methylamino)-1-(4-methylphenyl)propan-1-one		2.39	Stimulant
MDMA	1-(Benzo[d][1,3]dioxol-5-yl)-*N*-methylpropan-2-amine		2.28	Stimulant Euphoric Emphatogen

^a^values obtained from ChemSpider Database

Effluent samples were collected from the Plaszow WWTP, Krakow, Poland. It treats approximately 165,000 m^3^ of urban wastewater per day, which is over 70 % of the total volume of the city’s wastewater. Effluent water was collected after the secondary treatment, which involves primary settling, biological treatment and secondary sedimentation.

Effluent samples were collected in May 2012. Four wastewater samples (5 L each) were collected once a week, on Sunday. Equal aliquots of wastewater were taken every hour over a 24 h period, collected in pre-cleaned polyethylene containers with UV protection and stored at 4°C until the collection process was finished. Then, samples were transported to the laboratory and processed within 12 h. In the first step, before the solid phase extraction, wastewater was filtered using MN GF-4 and MN GF-1 glass fibre filters from Macherey–Nagel (Düren, Germany). Afterwards, samples were acidified to pH 4.5 with 2 M hydrochloric acid.

Oasis HLB 3 cc (60 mg/3 mL) extraction cartridges from Waters (Milford, MA, USA) were used in the analytical procedure. SPE was performed using a 12-port vacuum extraction manifold (J.T. Baker, Philipsburg, USA). The extraction cartridges were conditioned by 2 mL MeOH/NH_4_OH (v/v, 95:5) and 2 mL deionised water adjusted with hydrochloric acid solution to pH 4.5. 500 mL of samples adjusted to pH 4.5 were spiked with 4 µL (200 ng) internal standard solution of concentration 50 µg mL^−1^ before being passed through SPE cartridges. A flow of 5 mL min^−1^ water through the cartridge was achieved by applying a mild vacuum to the extraction manifold. The respective cartridges were then washed by 10 mL deionised water (pH 4.5) and completely dried under vacuum. The analytes were eluted twice from the cartridges with 2 mL of MeOH/NH_4_OH (v/v, 95:5). Extracts were pooled to 5 mL vials and stored in a freezer in temperature −24°C for 24/48 h until analysis. Directly prior to analysis 100 µL (0.025 M) hydrochloric acid were added to extracts. Subsequently, extracts were evaporated to 100 µL by nitrogen stream in a Pierce Reacti-Vap III evaporator and were finally reconstituted with mobile phase to a volume of 1 mL. The supernatants were successively transferred into 2 mL auto sampler vials for analysis by means of LC–ESI-QTOF-MS. In the experiments, all calibration solutions as well as extracts were dissolved in starting mobile phase (0.1 % formic acid in 95 % water/5 % acetonitrile).

LC/MS analyses were carried out using an Agilent Technologies 1200 Series liquid chromatography instrument coupled with a 6520 Accurate-Mass Q-TOF LC/MS detector equipped with electrospray ionization (ESI) manufactured by the same company. The mobile phase was composed of a mixture of 0.1 % (v/v) formic acid in water (A) and in acetonitrile (B). The separation was performed at 35°C using an Ascentic Express C18 column (7.5 cm × 2.1 mm × 2.7 µm) from Supelco. The flow rate was 0.3 mL min^−1^, and the volume of injection was 10 µL. All analyses were carried out in a gradient mode (shown in relation to content of phase B): 0 min—5 %, 16 min—20 %, 16.2 min—5 %, 21 min—5 %.

The instrument operated with an electrospray ion source (ESI) in positive ionization mode. Nitrogen was used as the drying gas at a temperature of 300°C and a flow rate of 10 L min^−1^ and the nebulizing gas at a pressure of 45 psi. Capillary voltage was set at 3000 V and skimmer voltage at 65 V. Fragmentor voltage was set at 120 V. The quadrupole was used as an ion guide in MS mode, and for selection of precursor ions with Δm/z = 1.3 in MS/MS mode. Nitrogen was used as the collision gas in MS/MS mode and collision energy was set at 4 V for MDMA and MDMA D5, 8 V for mephedrone and 4-MEC, 16 V for mCPP, 24 V for MDPV. Collision energy for compounds was obtained by using Mass Hunter Optimizer Software, Version B.02.00 (Agilent Technologies).

The mass range was 50–1000 m/z, in both MS and MS/MS modes. Spectra were internally mass-corrected in real time using an automatically introduced reference mass solution containing two compounds: purine ([M + H]+ = 121.0509) and HP-921—hexakis(1H,1H,3H-tetrafluoropropoxy)phosphazine ([M + H]+ = 922.0098).

The concentrations of target compounds were determined by using the main product ion (quantifier) presented in Table 2. Concentrations in the samples were calculated by comparing the peak area ratios of the analytes and surrogate standard to the corresponding ratios in the standard solutions. The stimulant drugs stock solution with a concentration of 10 µg mL^−1^ in methanol was used for preparation of calibration solutions. Calibrations curves were obtained from this stock solution spiked in mobile phase at 5, 10, 25, 50, 100, 250, 500, 1000 ng mL^−1^. All calibration solutions contained 200 ng mL^−1^ of the internal standard, MDMA D5.

**Table 2:** Chromatographic and mass spectrometric characterization of target compounds

Compounds	RT [min]	Precursor (m/z)	Product 1 (Quantifier) (m/z)	Product 2 (Qualifier) (m/z)
MDMA D5	3.614	199.1489	165.0874	136.0494
MDMA	3.88	194.1176	163.0749	105.0709
MPD	4.934	178.1226	160.1116	145.0882
4-MEC	6.571	192.1383	174.1275	146.0961
mCPP	8.095	197.0840	154.0418	119.0731
MDPV	10.639	276.1594	126.1278	135.0442

*Product 1* main product ions (quantifier), *Product 2* secondary product ions (qualifier)

*RT* retention time in min, precursor and product mass fragments

## Results and Discussion

Extraction efficiency was tested by placing 500 mL deionized water adjusted to pH 4.5 in volumetric flasks and then spiking them with prepared drugs stock solutions. After manual shaking, the samples were extracted by SPE. For each sample three extractions were prepared and each extract was analysed in triplicates. To check for concentration dependencies of the recovery rate and to determine the limit of quantification from the recovery experiment, deionized water samples were spiked to 40, 200 and 600 ng L^−1^ of the target compounds. All the samples were spiked with labelled internal standard as well as hydrochloric acid to adjust the pH to 4.5. The eluates were concentrated and reconstituted with mobile phase to 1 mL and extracts were analysed by LC–ESI–QTOF–MS. The results presented in Table 3 show that most recovery rates were between 50 % and 100 %.

**Table 3:** Recoveries (%) of the spiked samples by Oasis HLB cartridge

Spiked concentration in H_2_O	mCPP	4-MEC	MDPV	MPD	MDMA
40 ng L^−1^	61	63	81	39	43
	72	63	84	47	43
	66	66	79	40	50
200 ng L^−1^	100	62	110	37	45
	93	62	107	39	47
	90	59	104	39	47
600 ng L^−1^	99	59	117	39	46
	87	57	106	37	44
	104	63	131	40	49
Average recovery rate (%)	86	62	102	39	46
RSD %	17	4	16	7	5

The recovery rates were quantitated and the relative standard deviations (RSD  %) were about 10 %. The applied conditions of the liquid chromatograph allowed for the successful separation of all analytes within 11 min. Before analysis of target compounds, blank measurements were applied for detection of possible impurities. For all analytes, molecular ions were selected as precursor ions. Specific and intense product ions of each target analyte were used for quantification and a secondary product ion was used as qualifier ion for confirmatory purposes.

Details of the specific parameters for detection of the analytes are given in Table 2. Delta retention time in MS/MS mode was 3 min, the time window wherein the fragmentation occurs, i.e. retention time ±1.5 min.

Calibration curves were produced by a weighted (1/x) linear least square regression. Relative peak areas (ratios of the analytes to the surrogate standard) were used for calculations. Correlation coefficients, R, were in the range of 0.9982–0.9997. Limits of detection (LOD) and quantification (LOQ) were calculated on the basis of signal to noise ratios (S/N) of 3 and 10, respectively. All parameters are shown in Table 4.

**Table 4:** Linear ranges, correlation coefficients (R) of the calibration curves, sample based limits of detection (LOD) and limits of quantification (LOQ)

	Linear range (ng L^−1^)	R	LOD (ng L^−1^)	LOQ (ng L^−1^)
mCPP	5–1000	0.9996	2.2	6.6
4-MEC	5–1000	0.9995	2.7	8.8
MDPV	10–1000	0.9994	5.8	17.5
MPD	5–1000	0.9992	2.0	6.1
MDMA	5–1000	0.9997	2.3	6.9

The analytical results corrected for the average recovery (shown in Table 3) are shown in Table 5. The highest concentration found in wastewater samples corresponds to mephedrone (42.9 ng L^−1^). The detected concentrations of MDMA and 4-MEC were similar, ranging between 21.6 and 41.3 ng L^−1^. As the analytes concentrations in wastewater samples were low, the results were recalculated for recoveries at low concentration level (40 ng L^−1^), but the differences were insignificant.

**Table 5:** Concentrations in ng L^−1^ of psychoactive compounds refer to the weekly sampling intervals in WWTP effluents

Compound	Concentrations (ng L^−1^)			
	Week 1	Week 2	Week 3	Week 4
mCPP	<LOD	<LOD	<LOD	<LOD
4-MEC	35.3 ± 0.7	36.7 ± 2.8	41.3 ± 0.9	29.1 ± 1.4
MDPV	<LOD	<LOD	<LOD	<LOD
MPD	42.9 ± 4.6	29.0 ± 4.7	31.9 ± 1.6	21.6 ± 2.4
MDMA	40.7 ± 4.7	40.9 ± 1.2	31.9 ± 1.6	21.6 ± 2.4

MDPV and mCPP were not detected in the tested wastewater samples.

Loads (mg 1000 inhabitants^−1^ day^−1^) of illicit drugs discharged via effluents into the aquatic system were estimated from concentrations of each compound detected in the effluents (ng L^−1^) and the daily flow of effluents discharged to the aquatic system. Loads of illicit drugs (MDMA, mephedrone and 4-MEC), discharged by the WWTP ranged from 3.6 to 6.7 mg day^−1^ 1000 inhabitants^−1^ of MDMA, 3.6 to 7.1 mg day^−1^ 1000 inhabitants^−1^ of mephedrone and 4.8 to 5.8 mg day^−1^ 1000 inhabitants^−1^ of 4-MEC.

The hazard quotient (HQ) method was used to screen the toxicological risk level (Mendoza et al. 2014). The lack of aquatic ecotoxicological data for psychoactive compounds makes it difficult to conduct proper environmental risk assessment. For MDMA, Mendoza et al. (Mendoza et al. 2014) published a PNEC of 0.216 µg L^−1^ based on the lowest median lethal (effective) concentration (L(E)50) in algae, cladocerans and fish divided by an assessment factor of 1000. PNEC derived by the Ecological Structure Activity Relationships (ECOSAR) modeling. For mephedrone and 4-MEC, no PNECs could be found in available literature. The calculated HQs for MDMA, which are well below 1, up to 0.188, mean that, as far as data are available, the environmental risk is low but a potential adverse effect could be expected for MDMA. The values of HQs estimated in this work for an environmental risk assessment are in agreement with data reported by Mendoza et al. (Mendoza et al. 2014) and Bijlsma et al. (Bijlsma et al. 2014). These data were calculated in the effluent of the WWTP, so this is the worst case scenario because effluents are diluted further in a surface water.
